# Sample Preparation for Mass Spectrometry Imaging of Plant Tissues: A Review

**DOI:** 10.3389/fpls.2016.00060

**Published:** 2016-02-10

**Authors:** Yonghui Dong, Bin Li, Sergey Malitsky, Ilana Rogachev, Asaph Aharoni, Filip Kaftan, Aleš Svatoš, Pietro Franceschi

**Affiliations:** ^1^Biostatistics and Data Management, Research and Innovation Centre - Fondazione Edmund MachS. Michele all'Adige, Italy; ^2^Department of Plant and Environmental Sciences, Weizmann Institute of ScienceRehovot, Israel; ^3^Department of Chemistry and Beckman Institute for Advanced Science and Technology, University of Illinois at Urbana-ChampaignUrbana, IL, USA; ^4^Research Group Mass Spectrometry/Proteomics, Max Planck Institute for Chemical EcologyJena, Germany

**Keywords:** mass spectrometry imaging, molecular histology, MALDI, DESI, SIMS, plant metabolites

## Abstract

Mass spectrometry imaging (MSI) is a mass spectrometry based molecular ion imaging technique. It provides the means for ascertaining the spatial distribution of a large variety of analytes directly on tissue sample surfaces without any labeling or staining agents. These advantages make it an attractive molecular histology tool in medical, pharmaceutical, and biological research. Likewise, MSI has started gaining popularity in plant sciences; yet, information regarding sample preparation methods for plant tissues is still limited. Sample preparation is a crucial step that is directly associated with the quality and authenticity of the imaging results, it therefore demands in-depth studies based on the characteristics of plant samples. In this review, a sample preparation pipeline is discussed in detail and illustrated through selected practical examples. In particular, special concerns regarding sample preparation for plant imaging are critically evaluated. Finally, the applications of MSI techniques in plants are reviewed according to different classes of plant metabolites.

## Introduction

Mass spectrometric imaging (MSI) enables label-free detection and mapping of a wide range of molecules at complex surfaces, and it has become an attractive molecular histology tool in pharmaceutical and medical research. Since 2005 MSI has been gradually applied in plant research (Imai et al., 2005; Mullen et al., 2005). The information on the spatial organization of proteins and metabolites will greatly improve our understanding of plant metabolism and the biochemical functions of specific plant tissues (Lee et al., 2012; Matros and Mock, 2013).

**Figure 1:**
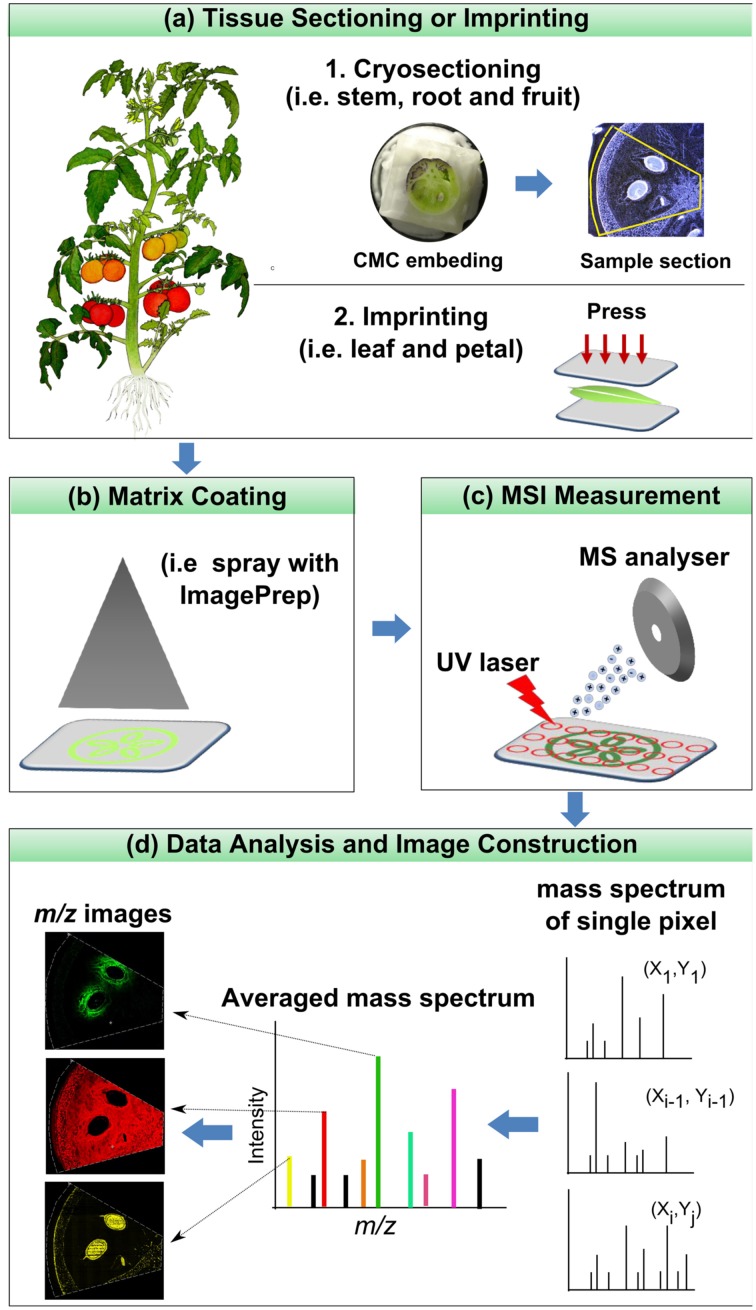
**Typical workflow of a MALDI imaging experiment**.

The core of MSI experiment is the mass spectrometer, which consists of three major parts: ion source, mass analyzer and detector. In the ion source, analytes are desorbed and ionized; in the analyzer, they are separated on the basis of their mass to charge ratios (*m/z*); and, finally, the separated ions are detected in the detector. As a final output a mass spectrum is generated by displaying the intensity of the detected ions over a full *m/z* scale. The basic principle of microprobe MSI is straightforward: the instrument collects a series of mass spectra by rastering a certain area of a tissue sample. The distribution maps of the analytes over the sample surface are subsequently generated by plotting the intensity of their individual *m/z* peak in the mass spectra against the *x-y* coordinate (Goodwin et al., 2008; Svatos, 2010). The typical workflow of a MALDI imaging experiment is shown in Figure 1.

Several ion sources are available for MSI, among which secondary ion mass spectrometry (SIMS), matrix-assisted laser desorption ionization (MALDI) and desorption electrospray ionization (DESI) are the most popular (Amstalden van Hove et al., 2010; Svatos, 2010). Many other MSI sources are still emerging, and they are usually derivatives or modifications of the above mentioned approaches. Among them it is worth mentioning laser ablation electrospray ionization (LAESI), laser ablation inductively coupled plasma (LA-ICP), liquid extraction surface analysis (LESA), direct analysis in real time (DART), and nano-desorption electrospray ionization (Nano-DESI). The different ion sources differ greatly in the method of ion generation (and hence on the nature of the produced ions), pressure regime, spatial resolution, probing depth, and speed. Each of them has its own advantages and disadvantages; for a comprehensive overview on this topic the reader is kindly referred to the following papers (Bhardwaj and Hanley, 2014; Boughton et al., 2015; Li et al., 2015; Sumner et al., 2015).

Sample handling is the most crucial step in MSI, as only an appropriate sample preparation can maintain the origin, distribution and abundance of the molecules, ensuring high-quality signals and sufficient spatial resolution (Grassl et al., 2011; Kaspar et al., 2011; Peukert et al., 2012; Spengler, 2014). Although Sample preparation for proteins and peptides has been somewhat standardized (Goodwin et al., 2008), there are very few examples of protein or peptide imaging in plant-based research (see Section Proteins and Peptides). Conversely, a large percentage of publications have dealt with plant secondary metabolites. The challenges are not only relevant to secondary, but also primary metabolites which can be oxidized, can diffuse through tissues during preparation, or be degraded through enzymatic processes or through exposure to light, heat or atmosphere (Lee et al., 2012).

MSI sample preparation methods for plant tissues are more challenging than those for mammalian tissues (Boughton et al., 2015; Heyman and Dubery, 2015). Cuticles in higher plant bodies represent the first barrier for direct MS imaging of internal metabolites, since it is difficult for most soft ionization techniques such as MALDI and DESI to penetrate through them (Thunig et al., 2011). Plant cell walls form the second barrier for MS imaging of molecules within the cell. For instance, when a matrix solution is sprayed onto the surface of a plant body, the cell wall prevents the solution diffusing across the cell wall, leading to the reduced “analyte extraction” efficiency in MALDI imaging (Takahashi et al., 2015). The high water content in plant tissues poses another challenge during cryosectioning. Plant tissues are more fragile upon freezing, and additionally, it is more difficult to get thin and intact sections for water-rich plant samples. Sample shrinkage or partial flaking is often observed upon dehydration, and this phenomenon usually results in an uneven surface which further affects MSI analysis. It should be noted that the sample shrinkage could also lead to mismatch between the MS image and the optical image (Cha et al., 2008), making biological interpretation difficult. This review therefore discusses the sample preparation strategies for plant tissues, aiming at providing guidelines for researchers who are planning to image different plant tissues and metabolites. In the first part, all the essential steps of sample preparation as well as specific considerations for plant tissues are discussed in detail. In the second part, the application of MSI to the analysis of different classes of metabolites in plant samples is summarized and discussed referring to a selected list of publications.

## Sample preparation for plant tissues

In this section we discuss each sample preparation step, including tissue storage (*2.1*), sectioning (*2.2*), mounting (*2.3*) and ionization supporting treatments (*2.4*) (Pol et al., 2010; Goodwin, 2012). In addition, other considerations for plant sample handling (*2.5*) such as the use of fresh sample or dry sample (*2.5.1*), removal of plant cuticle (*2.5.2*), matrix effects (*2.5.3*), and morphological effect (*2.5.4*) are also covered here. It is worth noting that sample preparation steps vary depending on the MSI instrument, on the nature of the tissues and on the analytes to be imaged.

### Sample storage

Samples are typically flash frozen in liquid nitrogen to prevent enzymatic degradation or analyte migration, and then stored at −80°C for up to 1 year with no reported significant degradation (Schwartz et al., 2003). However, water-rich plant samples may shrink upon long-term storage due to the gradual sublimation of water. When samples are too large and the storage space is limited, plant samples can be stored as section slides (Dill et al., 2011). For example, in our laboratory, several apple sections are mounted onto the glass slide, vacuum dried (~50 Torr, 4 h), vertically placed in a back-to-back manner into a 50 ml falcon/centrifuge tube with several small holes (~2 mm) drilled on its cap (i.e., corning® 50 mL PP centrifuge tubes, Sigma Aldrich). The tubes are subsequently vacuum sealed in a vacuum bag, and stored at −80°C. Vacuum sealing prevents the sample from contacting with air and water, and placing the section slide into the tube prevents the damaging of the section as it avoids direct contact with the bag during storage. When ready for use, sections can be recovered for 2 h under vacuum (~50 Torr). Our recent MALDI imaging study on the distribution of flavonoids in apple suggests that there are no significant quantitative differences in detection between long-term-stored (9 months) and fresh-prepared apple sections (Franceschi and Wehrens, 2014). A possible alternative is to preserve the tissue section as imprints on flat surfaces like PTFE sheet (Cabral et al., 2013) (details about imprinting are discussed in Section Removal of the Plant Cuticle). Imprinting is a method which can be useful for samples that could readily wither and fade even when stored under low temperature, as, for example, leaves and flowers, but the effect of storage on the quality of the imprints has to be still evaluated.

### Sectioning

**Figure 2:**
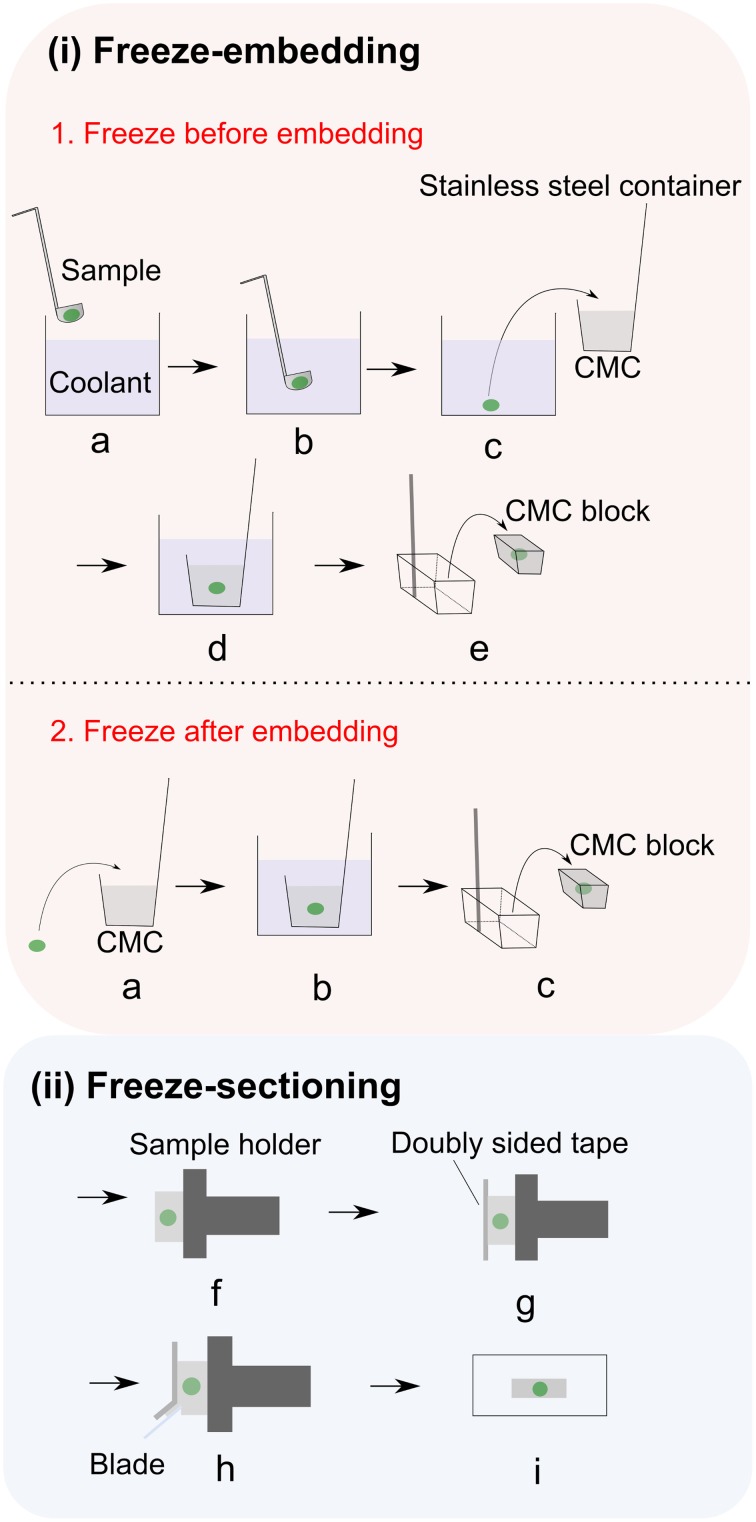
**Adhesive film assisted CMC embedding method. (i)** Freeze embedding can be achieved by either **(1)** freezing before embedding or **(2)** freezing after embedding. In freezing before embedding method: **(a,b)** Freezing of the sample in the coolant; **(c)** freeze-embedding of the frozen sample with 4–5% CMC gel; **(d)** completely freezing the CMC gel in coolant (i.e., hexane); **(e)** removing the CMC block from container;. In freezing after embedding method: **(a)** placing the sample into 4–5% CMC gel; **(b)** completely freezing the CMC gel in coolant (i.e., hexane); **(c)** removing the CMC block from container; **(ii**) After freeze embedding, the CMC block is subjected to freeze sectioning: **(f)** fixing the embedded sample to the sample holder with CMC gel and then attaching it to the cryomicrotome; **(g)** adhering the film to the exposed cutting surface; **(h)** cutting the sample; **(i)** placing sample section on the glass slide. Figure is adapted with permission from Kawamoto (2003).

Plant cells have rigid cell walls, large intercellular spaces and they are often rich in water, thereby embedding materials are often used in conventional plant histology practice to maintain the tissue morphology and ensure precise sample sectioning. Unfortunately, many of the commonly used embedding media are incompatible with MSI. For example, optimum temperature cutting (OCT) compound, which is a mixture of polyethylene glycols, is strongly discouraged since its use has been associated with diffusion into tissue and smearing across the surface during sectioning (Zaima et al., 2010b). Carboxymethyl cellulose (CMC) (Goto-Inoue et al., 2012), gelatin (Chen et al., 2009; Gemperline et al., 2015), ice (Khatib-Shahidi et al., 2006; Gorzolka et al., 2014) or their combinations (Nelson et al., 2013) have been successfully employed as MSI-compatible embedding mediums. Zaima and coworkers found that with the assistance of adhesive film, CMC embedding offers good sectioning performance (Zaima et al., 2010a; Yoshimura et al., 2012b). The use of adhesive film reduced the distortion of the sections and the dislocation of the analytes, additionally facilitating the transfer and attachment of sections to the slides. This method is described in Figure 2 (Kawamoto, 2003). It is important to mention that this method is initially proposed for histology and tissue staining so steps such as fixation, washing and staining should be avoided in MSI.

Cryosectioning is the most commonly used method to prepare plant tissue slices as freezing well quenches metabolic processes. Before sectioning, samples are generally snap frozen in various low temperature conditions such as inside an ultra-low temperature freezer, or in powdered dry ice, liquid nitrogen or liquid nitrogen-chilled isopentane (Zaima et al., 2010b). Liquid nitrogen freezing usually makes plant sections brittle, and it results in ice crystal formation, thus rapid plunging of the tissue into liquid nitrogen is not recommended. Floating tissues in aluminum foil in liquid nitrogen (Schwartz et al., 2003; Nimesh et al., 2013) or freezing tissues on dry ice-chilled steel plate is more favored. Dry tissues like plant stems, can be also sectioned at room temperature (RT) using a microtome (Imai et al., 2005; Saito et al., 2008, 2012; Lunsford et al., 2011; Zhou et al., 2011) or vibratome (Lunsford et al., 2011). Sectioning at RT is only limited to some dry/dead plant tissues such as wood with dense and relatively hard structure. This is not suitable for most fresh tissues due to the dispersing of plant juice. Additionally, the enzyme-substrate reaction may still be active when tissues are sectioned at RT.

Section thickness is another important parameter to take into account because it affects the number and the intensity of the ion signals in MSI instruments. For example, poor ion intensity due to inefficient ionization can be expected in MALDI instruments where the “z” direction of the sample holder is not easy to adjust or the laser cannot be focused to the top of the thick tissue section. In addition, electrical non-conductivity (i.e., in non-orthogonal TOF-MS), high impurities, matrix absorption (in porous tissues) and tissue distortion (i.e., in linear/reflectron MALDI-TOF) in thick sections could also lead to the reduced signal intensity (Sugiura et al., 2006).

For mammalian tissues, section thickness between 5 and 20 μm is recommended for analysis of low molecular weight molecules, and < 5 μm thickness for high molecular weight proteins (*m/z*>9000) (Sugiura et al., 2006). In contrast, the relationship between tissue thickness and the quality of MS spectrum has been seldom studied in the MSI of plant sections. Generally it is difficult to cut thin slices of water rich plant tissues due to crumbling and fracturing of the tissues. The thickness of most plant sections in current MSI studies is about 50 μm, providing a good compromise between optimum MSI performance and practicality, especially when a large number of samples have to be prepared (Peukert et al., 2012).

### Mounting

Sample tissues can be either mounted onto a glass slide or on a MS-compatible plate (Schwartz et al., 2003). Different mounting surfaces are required depending on the ionization technique. For non-orthogonal MALDI TOF, conductive surfaces such as steel and metal-coated glass slides are required, while, for orthogonal MALDI TOF normal glass slides are sufficient. In contrast, in ambient ionization techniques like DESI, non-conductive surface is used in order to avoid the neutralization of charged spray solvent (Takats et al., 2005; Costa and Cooks, 2007, 2008).

Three mounting approaches are commonly used to attach plant sections onto the glass slide: the sample can be either secured by using double sided tape/epoxy glue or it can be thaw-mounted. The use of double sided tape is fast and easy, but care should be taken not to contaminate the sample. Epoxy glue is suitable for delicate samples and it does not produce extra mass signals (Kaftan et al., 2014). Thaw-mounting is usually used to attach tissue sections acquired by cryosectioning. This approach minimizes the risk of sample contamination, but relocation of water soluble analytes resulting from water condensation during thaw mounting is a major concern (Cha et al., 2008). In the case of water rich plant tissues, it is necessary to consider not only the condensation of atmospheric water, but also water originating from the sample itself. With this specific method, downstream sample processing steps should be minimized to avoid washing sample off the glass slide by any vigorous solution-based treatments (i.e., the washing steps in MSI of proteins for the purpose of removing, e.g., salts and lipids) (Goodwin et al., 2008). To alleviate this problem, thaw mounted samples are usually dried within a desiccator at a reduced pressure (Lee et al., 2012; Boughton et al., 2015).

### Ionization aiding treatments

As already discussed, molecules have to be ionized before MSI analysis, and in some case the ionization efficiency and its selectivity can be increased by using specific ionization aiding treatments. In SIMS, the ionization is direct: a focused high energy primary ion beam (e.g., Ar^+^, Ga^+^, In^+^) is used to strike the sample surface. The analyte molecules are then released from the surface and ionized upon collision with the primary ions (Amstalden van Hove et al., 2010). The high energy used in SIMS usually causes extensive secondary ion fragmentation, limiting its practical mass range to ~ m/z 1000 (Heeren et al., 2006). Recently, several strategies aiming at extending the potential of SIMS and at increasing the ionization efficiency of large intact biomolecule have been proposed, one of them is to coat the sample surface with common MALDI matrices, possible alternatives are metallization of samples with silver and gold (Delcorte et al., 2003; Altelaar et al., 2006).

In MALDI, the deposition of a matrix over the sample surface serves several functions, in particular: (i) extraction of analytes from the sample surface, (ii) co-crystallization of analytes and matrix, and (iii) absorption of the laser energy aiding desorption of the molecules from the surface into the gas phase, where ionization eventually occurs (Lewis et al., 2006). Earlier on, MALDI imaging was almost exclusively performed using conventional matrices such as α-cyano-4-hydroxycinnamic acid (CHCA) and 2,5-dihydroxybenzoic acid (DHB). However, the imaging of small molecules, especially those with molecular weights similar to the one of the matrix, is problematic due to the high degree of ion suppression caused by the presence of the dominant matrix ion signals. A variety of alternative matrices have been proposed to obviate this problem. The first possibility is to use ionless matrices which do not produce matrix-related interfering ions, i.e., 1,8-bis(dimethylamino) naphthalene (DMAN) (Shroff et al., 2009). However, it is important to point out that DMAN is instable in the high-vacuum MALDI (Thomas et al., 2012), which limits its application only in atmospheric pressure (AP)-MALDI. Alternatively, it is possible to choose high-molecule-weight matrices which do not generate ions in the low mass region [i.e., porphyrins (MW: 974.57) (van Kampen et al., 2009)]. Another possibility is to use inorganic matrices which show relatively clean background. This can be deposited over the sample surface (i.e., colloidal graphite, Zhang et al., 2007; Cha et al., 2008) or they can constitute the target plate, like in the case of electrochemically etched porous silicon (desorption/ionization on silicon MS, DIOS-MS) (Wei et al., 1999; Ronci et al., 2012). At the same time, it is also possible to use nanostructures coated with liquid-phase perfluorinated initiator molecules. This type of solution has been already used in LDI MS and NIMS (nanostructure initiator MS, NIMS) (Yanes et al., 2009).

**Figure 3:**
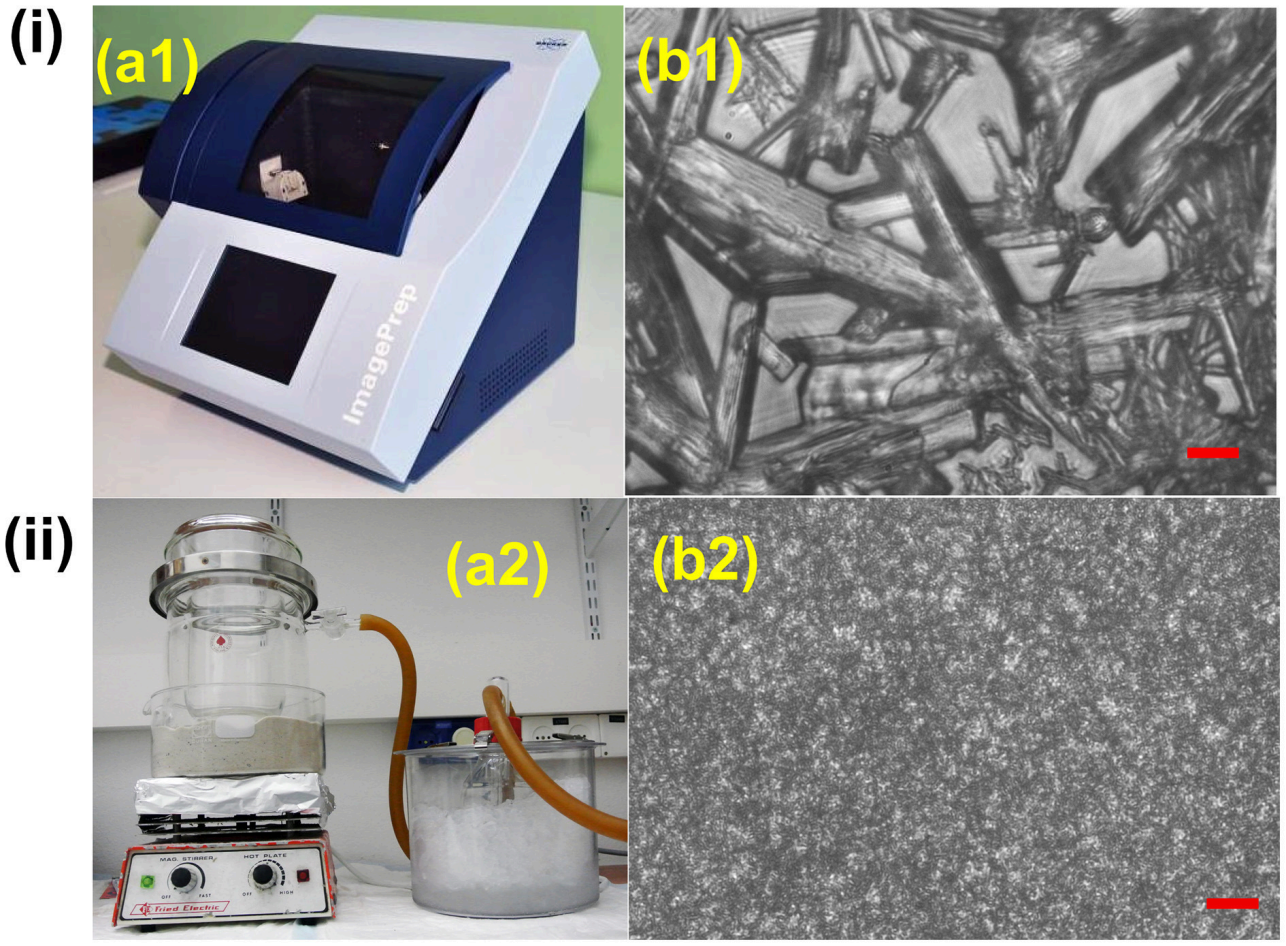
**Comparison between (i) spray based (Bruker ImagePrep system) and (ii) vacuum sublimated DHB matrix in MALDI imaging**. **(a1**) 30 gL^−1^ DHB (in 70% ACN solution) was used. Typical instrumental parameters include: 0.8 s spray, 10 s incubation, 70 s drying, and 60 spray cycles. **(a2)** 300 mg DHB was used. Major parameters include: ~120°C heating temperature, 0.05 Torr vacuum pressure, 10–11 min sublimation, matrix density 0.05 mgcm^−2^. **(b1,b2)**: DHB crystal sizes resulting from Bruker ImagePrep **(b1)** and sublimation **(b2)**, scale bar: 10 μm. Figures were adapted with the permission from Ref. (Hankin et al., 2007; Franceschi et al., 2012).

MALDI matrix can be deposited on tissue sections in several ways. The most common methods include spraying, spotting, sublimation and dry coating. Spraying can be performed manually, i.e., with an airbrush, or automatically, using commercial devices like ImagePrep (Bruker) (Figure 3i) or TM-Sprayer™ (HTXImaging). Manual spraying requires skill because inhomogeneous matrix application and analyte delocalization can easily occur, while automatic solutions are more reproducible. In spotting, the matrix can also be delivered by micro-spotting to a specific sample surface location with robots like the CHIP-1000 chemical printer (Shimadzu). This method allows accurate deposition of matrix onto a tissue section, and this is good to attain a good quantitative MS signal generation in MSI (Vegvari et al., 2010). Sublimation allows fast and uniform matrix deposition (Figure 3ii). During sublimation, the matrix is placed inside a sublimation chamber, and the sample plate with the tissue is placed inverted over the top of the matrix and attached to a cold-finger (Norris and Caprioli, 2013). The matrix is heated at elevated temperature and under reduced pressure, while the sample itself remains cooled as heat could degrade the analytes. Sublimation is solvent free, therefore diffusion of most analyte molecules during matrix application is almost eliminated, even though it has been reported that lipids from very fatty tissues can to some degree diffuse by capillarity through dry matrix during storage (Berry et al., 2011). It is important to point out that, owing to the absence of solvent, the extraction efficiency can be poor for some compounds like peptides and proteins. Additional re-hydration has been demonstrated to improve the detection sensitivity (Bouschen et al., 2010; Yang and Caprioli, 2011). Other advantages of sublimation include high reproducibility, increased matrix purity, formation of fine matrix crystals and relatively low cost (Hankin et al., 2007). Finely ground matrices and nanomaterials can be also spread over the sample surface by using a fine mesh sieve (i.e., 20–50 μm) (Puolitaival et al., 2008; Chaurand et al., 2011). This method is simple, fast and meanwhile it avoids analyte delocalization, but also in this case the analyte extraction efficiency is reduced.

As far as DESI is concerned, the first element determining the ionization efficiency is the composition of the spray solvent, which should be optimized taking into account the metabolites under investigation and the specific characteristics of the samples (Badu-Tawiah et al., 2010; Green et al., 2010). In general, a higher fraction of water is used to have long lasting signals, while a higher proportion of methanol is used when higher spatial resolution is required (Manicke et al., 2008). When necessary, a reactive reagent can also be added to the spray solvent to selectively improve the ionization efficiency of analytes which are difficult to ionize under normal DESI conditions (Zhang and Chen, 2010; Muller et al., 2011; Lostun et al., 2015).

### Other considerations for plant samples preparation

#### Fresh sample vs. dry sample

Fresh plant tissues would be ideal for MSI studies since they are chemically unmodified and treatment-free. In these conditions, the spatial arrangement of the molecules is preserved and the risk of chemical contamination during sample handling is minimized. Fresh tissues, however, can be directly analyzed only by using ambient ionization techniques, while they are likely to shrink during MSI analyses performed under vacuum (e.g., MALDI or SIMS). Sample shrinkage could result in mismatch between the MS image and the optical image (Cha et al., 2008), making biological interpretation difficult. Moreover, shrinkage during MSI analysis may cause unwanted mass shift during MSI analysis in non-orthogonal TOF-MS analyzer (as the flying time can be different for the same analyte located on an uneven surface), hampering molecule identification and reproducibility (Kulkarni et al., 2015). Another concern is that inside fresh samples the biological processes are still active and they may cause degradation and/or chemical modification during the analysis (Cha et al., 2009). In the case of infrared (IR)-MALDI and LAESI, since native water from the sample is employed as a matrix, the sample tissues have to be fresh or at least not totally dried out (Li et al., 2007a,b; Shrestha et al., 2011).

Most plant samples are vacuum- or freeze- dried prior to MSI. Vacuum desiccation can be applied for thin plant organs, such as leaves and flowers (Cha et al., 2008, 2009; Li et al., 2011; Korte et al., 2012), or for microtome or razor blade-sectioned samples, such as apple section (Franceschi et al., 2012). Vacuum pressure and drying time should be carefully optimized according to the nature of the sample and analytes of interest. It is likely, for example, that some compound (i.e., volatile essential oils, terpenes, alcohols and other small molecules) will be lost during vacuum desiccation, but the majority of molecules are expected to be unaffected due to their low vapor pressure (Franceschi et al., 2012). This has been confirmed by comparing MS profiles of target metabolites between fresh and dried *Arabidopsis* samples (Cha et al., 2008).

#### Removal of the plant cuticle

**Figure 4:**
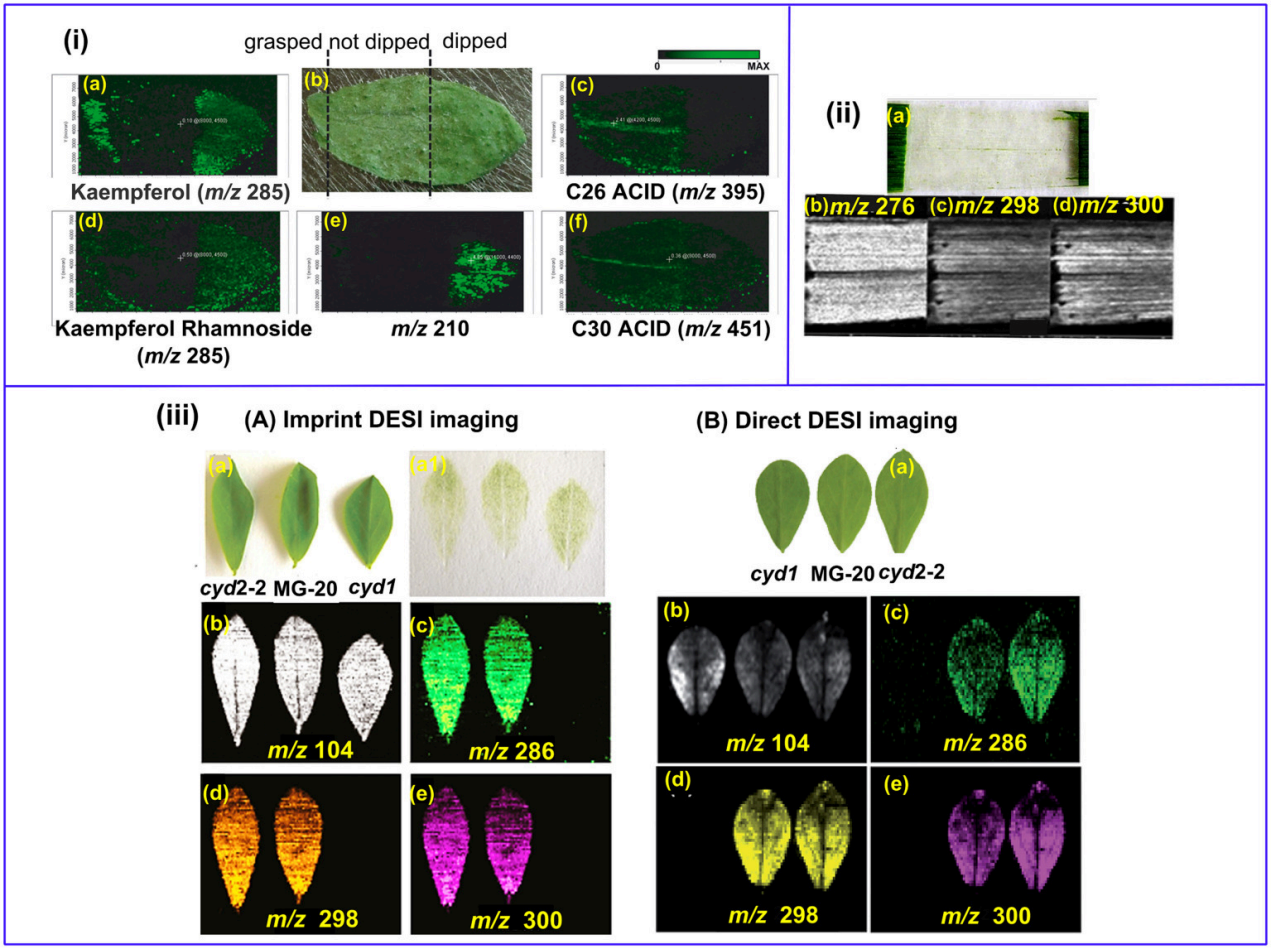
**MS imaging of metabolites below the cuticle. (i)** MALDI imaging of *Arabidopsis* leaf. The left area of the leaf was grasped using forceps, the middle part remained un-treated and the right area was chloroform-dipped **(b)**; Kaempferol **(a)** and Kaempferol Rhamnoside **(d)** were readily detected in the forceps- grasped and chloroform-dipped areas, while C26 **(c)** and C30 **(f)** fatty acids were washed off in the chloroform-dipped area. An un-identified analyte *m/z* = 210 **(d)** showed high abundance in the chloroform-dipped area. **(ii)** DESI images of the hydroxynitrile glucosides of *m*∕*z* = 276 **(b)**, 298 **(c)** and 300 **(d)** from barley leaf epidermis. The leaf abaxial epidermis strip was physically peeled off **(a)**. **(iii)** Comparison of imprinting and direct DESI imaging of *L. japonicus* leaves. **(A)** Indirect DESI imaging of the leaf imprint on Teflon. **(B)** Direct DESI imaging of the leaf, with chloroform, methanol and water (1:1:0.4, v/v/v) as spray solvent. **(a)** Optical image of *L. japonicus* leaves. **(a1)** Optical image of the leaf imprints on Teflon. **(b–e)** DESI images of *m/z* 104, 286, 298, and 300, respectively. Figures were adapted with the permission from Cha et al. (2008), Li et al. (2011, 2013), and Bjarnholt et al. (2014), respectively.

Land plant organs, such as leaves and flowers are covered with a lyophilic cuticular layer (0.1–10 μm in thickness) (Riederer and Schreiber, 2001). The cuticle serves for a variety of important biological functions, and MS imaging of plant cuticles is specifically discussed in Section Lipids and Fatty Acids. On the other hand, the cuticle poses a barrier for the MS imaging of the internal metabolites since soft ionization techniques such as MALDI and DESI cannot easily penetrate through it. LAESI-MSI is capable of depth profiling and it can be applied for imaging the internal metabolites, but its spatial resolution is still limited (typically 300 μm) (Nemes et al., 2009). Sample cuticle can be either physically removed or chemically washed off. For example, kaempferol and kaempferol rhamnoside were mostly detected in *Arabidopsis* leaf areas pre-treated with chloroform for 60 s (Figure 4ia,d,e) (Cha et al., 2008). However, those “aggressive methods” may delocalize and/or wash away the target compounds, as shown in the cases for C26 and C30 fatty acids (Figure 4ic,f). In addition, not all plant epidermis can be physically removed with ease as the in the case of barley leaf (Figure 4iia; Li et al., 2011), and scratching or grasping of the leaf cuticle will deter either the detection (Figure 4ie) or spatial resolution (Figure 4ia,c,d,f) of the analytes (Cha et al., 2008).

Imprinting is a promising way to circumvent this problem. With this approach, plant tissues are pressed onto porous Teflon (Li et al., 2011, 2013; Thunig et al., 2011), porous polytetrafluoroethylene (PTFE) (Muller et al., 2011), TLC plate (Cabral et al., 2013; Hemalatha and Pradeep, 2013), print paper (Ifa et al., 2011), or tape (Tata et al., 2014) by applying a moderate pressure over the plant tissues, thereby transferring the plant metabolites onto a flat hard surface while keeping their spatial distribution. Li et al. successfully imaged the distribution of hydroxynitrile glucosides in leaves of *L. japonicas* with both imprinting (Li et al., 2013) and direct DESI imaging (Bjarnholt et al., 2014). The abundance of hydroxynitrile glucosides were rather even in imprinting DESI imaging (Figure 4iiiAb–e), while direct DESI imaging showed a decreased abundance of hydroxynitrile glucosides in the leaf midvein, which was possibly due to the reduced accessibility to the solvent spray in direct DESI imaging (Figure 4iiiBb–e). A recent publication demonstrated that the transfer efficiency could be improved with the assistance of solvent extraction and/or heating during TLC-imprinting (Cabral et al., 2013). However, imprinting is only efficient for relatively “fleshy” plant tissues and the spatial resolution is limited since analytes can be smeared during imprinting (Lee et al., 2012).

#### Matrix effects

The chemical composition of the sample matrix can affect, in most cases negatively, the ionization efficiencies of the analytes of interest in MS analysis (this phenomenon is widely known as “matrix effect”). Although the limited sample pretreatment and the absence of separation are among the major advantages of MSI, they also make MS imaging prone to matrix effects, which alter the observed molecular distribution (Lanekoff et al., 2014). Matrix effects have been reported in SIMS (Jones et al., 2007), MALDI (Hankin et al., 2011; Janfelt et al., 2012; Wang et al., 2012), and nano-DESI imaging (Lanekoff et al., 2014). Since matrix effect is intrinsically associated with MS, it is also a major challenge in MSI of plant tissues even though it has not yet been well studied. Several strategies have been used to compensate for matrix effects in MSI, such as desalting the tissue sections prior to MSI analysis (Wang et al., 2012), and normalizing ion signals to the signals of their corresponding internal standards (Lanekoff et al., 2014).

#### Morphological effects

Similarly to what happens in the case of the matrix effects, the physical properties of the sample can also influence the analyte ionization process. As a consequence, variations in the physical properties of a heterogeneous tissue surface can affect the ion yield, resulting in MS images misrepresenting the real distribution of the metabolites. This phenomenon has been reported in a MALDI imaging study of tobacco root, where a notable loss of ion signals was observed in the central root region due to the reason that the MALDI matrix was mostly absorbed in that region (Peukert et al., 2012). It has been proposed that spraying the sample surface with large amounts of matrix could reduce the surface effects at least in MALDI (Lee et al., 2012). In DESI, the sample surface effect can be more obvious. It has been shown that both the chemical (i.e., bond strength and polarity) (Takats et al., 2005; Ifa et al., 2008; Manicke et al., 2008; Volny et al., 2008; Benassi et al., 2009; Douglass et al., 2012) and the physical properties (i.e., conductivity and roughness) (Takats et al., 2005; Manicke et al., 2008) of the surface strongly affect the DESI results. In particular, they impact on the lower limits of detection/quantification (LOD/LOQ) of the analytes, on the signal stability, on the degree of carryover, but also on the reproducibility and on the linear dynamic range. Additionally, it has been shown that the DESI source acts as a direct current capacitor and the surface properties play an important role in the charge transfer (redox) process (Volny et al., 2008; Benassi et al., 2009; Dong et al., 2015). Imprinting can be a promising way to minimize sample surface effect in DESI imaging, since the analytes are transferred to a homogeneous surface.

## Application of MSI in plants

Arguably, plant based MSI has historically been focused on methodological aspects (Matros and Mock, 2013), but now the technique has matured to a point where it has also been used to address biologically important questions (Lee et al., 2012) in fields like plant-environment interactions (Shroff et al., 2008, 2015; Klein et al., 2015; Ryffel et al., 2015; Soares et al., 2015; Tata et al., 2015), new compound identification (Jaeger et al., 2013; Debois et al., 2014), and functional genomics (Korte et al., 2012; Li et al., 2013). In the following section, we summarize the recent studies of MSI in plants organizing them on the bases of the class of molecules which were the subject of each specific study. The key characteristics of some selected applications are summarized in Table 1. Among the different aspects, we will focus on the choice of matrix for MALDI and of spray solvents for DESI since these elements are crucial in determining the quality of MSI analyses.

**Table 1:** **Selected examples of studies in which MSI was used to detect different classes of plant metabolites**.

**Chemical class**	**MSI Source (Ion mode)**	**Matrix or Spray solvent**	**Sample**	**Analyte**	**Sample preparation**	**References**
Carbohydrates	MALDI (+)	CHCA (in MeOH:H_2_O, 1:1, + 0.1% FA)	Wheat stem	Oligosaccharides	• Cryosecting (−20°C, 50 μm) and hand sectioning • Matrix applied with airbrush • Double sided tape mounting	Robinson et al., 2007
	MALDI (+)	DHB, CHCA, SA 20 mg/ml (ACN:H_2_O, 1:1, +0.1% TFA)	Poplar stem	Cellulose	• Cryosecting (−8°C, 50 μm) • Matrix applied with oscillating capillary nebulizer • Double sided tape mounting	Jung et al., 2010
	MALDI (+)	DHB, 25 mg/ml (in 0.05 mM aqueous sodium acetate)	Poplar stem	Cellulose hemicellulose	• Microtome sectioning (room temperature, 50 μm) • Matrix applied with Meinhard nebulizer • CryoJane tape mounting	Lunsford et al., 2011
	MALDI (+)	DHB/DMA, 100 mg/ml (in H_2_O/ACN/ DMA, 1:1:0.02)	Wheat grain	β-glucan arabinoxylan	• Vibratome sectioning (in 70% ethanol, 60 μm;) • Sections were stored in 70% ethanol at 4°C until analysis • Matrix applied with ImagePrep • Conductive carbon tape mounting	Veličković et al., 2014
	IR-MALDI (+)	Native water in the samples	Strawberry	Fructose/glucose sucrose	• Hand cutting (room temperature, 0.2–0.5 mm) • Fresh sample	Li et al., 2007b
Organic acids	GALDI (−)	Colloidal graphite (in 2-propanol)	Apple; Strawberry	Malic acid ascorbic acid citric acid quinic acid	• Cryosecting (liquid nitrogen pre-treated) • Colloidal graphite applied by air spray • Double sided tape mounting	Zhang et al., 2007
	DESI (−)	ACN:H_2_O (4:1)	Grape leaf petiole	Tartaric acid	• Hand cutting (room temperature, 0.3 mm) • Double sided tape mounting	Dong et al., 2015
Lipids and Fatty acids	MALDI (±)	DHB, 50 mg/ml (in MeOH:H_2_O, 7:3)	Black rice seed	Lysophosphatidylcholine, phosphatidylcholine, Phytic acid gamma-Oryzanol ahpha-Tocopherol	• Cryosecting (-80°C frozen section and freeze imbedded section with 2% CMC at -80°C, 8 μm) • Matrix applied with airbrush • Double sided tape mounting	Zaima et al., 2010a
	MALDI (+)	DHB	Cotton seed	Phosphatidylcholines, triacylglycerols, phospholipids	• Cryosecting (unfixed and paraformaldehyde fixed sections, 20°C, 30 μm) • Matrix applied via sublimation	Horn et al., 2012
	MALDI (−)	DAN	Maize leaf	Glycerolipids	• Cryosecting (freeze imbedded section with gelatin, 10 μm) • Matrix applied via sublimation	Korte et al., 2015
	LDI (+)	Colloidal silver	*A. thaliana* leaf & flower	Epicuticular wax	• Vacuum dried (~50 Torr, 30–60 min) • MicroFlow PFA-ST • Nebulizer • Doubly sided tape mounting	Cha et al., 2009
	MALDI (+)	Lithium-DHB, 20mg/ml (in acetone: dichloromethane, 9:1)	*A. thaliana* leaf; Date palm tree leaf	Wax esters	• Desiccator dried samples • Matrix applied with airbrush	Vrkoslav et al., 2010
Proteins and Peptides	MALDI (+)	Sinapic acid	Soybean cotyledon	Proteins	• Cryosectioning (10–15 μm)	Grassl et al., 2011
	MALDI (+)	Unknown	Barley grain	Proteins	• Unknown	Kaspar et al., 2011
	MALDI (+)	10 g/L CHCA: 10 g/LAniline (in ACN:H_2_O:TFA, 50:50:0.1)	Tomato fruit	Protein	• Cryosectioning (5% CMC embedding, 50 μm) • Matrix applied with ImagePrep and dried for 3 h	Bencivenni et al., 2014
Terpenoids	LDI (−)		Hypericum leaf, placenta, stamen and stylus	Naphthodianthrones	• Fresh sample or Cryosectioning (60 μm)	Holscher et al., 2009
	DESI (−)	MeOH:H_2_O, 1:1, +1% ammonium	Hypericum	Hyperforin Hypericin	• Imprinting on porous Teflon	Thunig et al., 2011
	DESI (−)	100 uM NH4Cl in MeOH	Red alga^*^	Bromophycolide A and B	• Preserved with 10% formalin in seawater and kept moist with seawater	Lane et al., 2009
Alkaloids	MALDI (+)	Saturated CHCA (in methanol)	Capsicum fruit	Capsaicin	• Cryosectioning (−20°C, 70 μm) • Matrix applied with airbrush • Thaw-mounted	Taira et al., 2012
	DESI (−)	MeOH:H_2_O, 9:1	*Myristica malabarica* seed	Malabaricone C	• Imprinting on a printer paper	Ifa et al., 2011
	MALDI (+)	DHB, 30 mg/ml (in MeOH:H_2_O, 1:1+1%TFA)	Fruiting bodies of *M*.metata^*^	6-Hydroxymetatacarboline D	• Freeze-dried • Matrix applied with ImagePrep • Double sided tape mounting	Jaeger et al., 2013
	LDI (−)		Tomato leaf	Tomatine	• Imprinting on pencil-lead-coated glass	Li et al., 2014b
Glycosides	MALDI (−)	9-aminoacridine 15 mg/ml (MeOH)	*A. thaliana* leaf	Glucosinolates	• Fresh sample • Matrix applied with airbrush • Double sided tape mounting	Shroff et al., 2008
	MALDI (−)	9-aminoacridine (neat)	*A. thaliana* leaf	Glucosinolates	• Fresh sample • Matrix applied via sublimation (170°C, 2 × 10^−3^ mbar) • Double sided tape mounting	Shroff et al., 2015
	DESI (+)	MeOH:H_2_O, 4:1	Cassava tubers	Gyanogenic glycosides	• Cryosectioning (50 μm) • Thaw-mounted and vacuum dried	Li et al., 2013
Phenolics	MALDI (+)	DHB, 50 mg/mL (in MeOH:H_2_O, 7:3)	Rabbiteye blueberry	Anthocyanins	• Cryosectioning (50 μm) • Matrix applied with airbrush • Thaw-mounted and air dried	Yoshimura et al., 2012a
	MALDI (+)	DHB, 50 mg/mL (in MeOH:H_2_O, 7:3)	Black rice seed	Anthocyanins	• Cryosectioning (freeze-embedded with 2% CMC at −80°C, 10 μm) • Matrix applied with airbrush • Double sided tape mounting	Yoshimura et al., 2012b
	GALDI (−)	Colloidal graphite (in 2-propanol)	*A. thaliana* leaf, flower and stem	Flavonoids	• Vacuum dried (for leaf and flower, ~50 Torr, 30 min) and cryosectioning (for stem)	Cha et al., 2008; Korte et al., 2012
	SIMS (Bi^3+^ ion beam)		Pea seed; *A. thaliana* seed	Flavonoids	• Cryostat sectioning (−20°C, 12 μm) for pea seed • Ultramicrotome sectioning (polyester resin embedding) for A. thaliana seed • Vacuum dried (few hectopascals 15 min)	Seyer et al., 2010
Element	LA-ICP		*Elsholtzia splendens* leaf	K, P, Mg, Mn,	• Fresh sample	Becker et al., 2008
	Nano-SIMS (Cs+ ion beam)		Rice node, internode and leaf sheath	^12^C^14^N^−^, ^28^Si^−^, ^56^Fe^16^O^−^, ^75^As^−^, ^32^S^−^, ^63^Cu^−^, ^31^P^−^, ^64^Zn^16^O^−^	• Samples were coated with 5 nm of platinum • Ultramicrotome sectioning (1 μm) • Sample coated with platinum	Moore et al., 2014
	SIMS (gallium ion beam)		Wheat grain	O^−^, ${\text{PO}}_{2}^{-}$, Mg^+^, Ca^+^, Na^+^, K^+^	• Ultramicrotome sectioning (polyester resin embedding)	Heard et al., 2002

DMA, N,N-Dimethylaniline; SA, sinapinic acid; DAN, 1,5-Diaminonaphthalene; CAN, acetonitrile; MeOH, methanol; MALDI, matrix assisted laser desorption ionization; LDI, laser desorption ionization; GALDI, graphite assisted laser desorption ionization; DESI, desorption electrospray ionization; SIMS, secondary ion mass spectrometry; LA-ICP, laser ablation inductively coupled plasma.

* Red alga Callophycus serratus and M. metata are not classified as plant.

### Carbohydrates

Carbohydrates are initially synthesized through photosynthesis in plants, and they are well known for their essential roles. Additionally, they could also function as signaling molecules, in a way similar to hormones (Trouvelot et al., 2014).

Distribution of carbohydrates has been mapped by MALDI in several plant samples (Robinson et al., 2007; Jung et al., 2010; Yoshimura et al., 2012a; Veličković et al., 2014) and in these applications DHB and CHCA are the most common matrices. DHB has proved to be slightly better than CHCA in detecting small oligosaccharides such as glucose and sucrose (Zhang et al., 2007). Colloidal graphite (Graphite assisted laser desorption ionization, GALDI) has been proposed as an alternative matrix for imaging small oligosaccharides since it largely reduces the matrix interference in the small mass region (*m*∕*z* < 500) (Zhang et al., 2007). IR-MALDI has also been used to image carbohydrates distribution in different plants, such as strawberry (Li et al., 2007b) and lily flower (Li et al., 2008). In MALDI imaging, carbohydrates are mostly detected in positive ion mode. Application of DESI imaging in localization of carbohydrates in plants is rarely reported (Thunig et al., 2011), partially due to the low selectivity and sensitivity of this technique toward this class of molecules. Yet 3-nitrophenylboronic acid and N-methyl-4-pyridineboronic have been suggested as effective reagents added to the DESI spray solvent for *in situ* derivatization of sugars (reactive-DESI) to improve the ionization efficiency of intact sugars in complicated biological matrices (Zhang and Chen, 2010).

### Lipids and fatty acids

Lipids are a major component of plant tissues. They are present in all cells as constituents of the various cellular membranes. Other lipids such as waxes, fulfill important protective functions both in plant leaves and fruits. Pigmented lipids are involved in light harvesting and energy transduction, while others are concerned in electron transport processes. Certain plant lipids also represent energy stores that can be mobilized and consumed for growth and development of plants (Harwood, 1996).

The localization of various unsaturated lipids has been mapped in rice (Zaima et al., 2010a) and cotton seeds (Horn et al., 2012), *Camelina sativa* seed and avocado fruit (Horn et al., 2013) using MALDI imaging. In these tissues, lipids are readily detected as multiple adducted ions (primarily H^+^, Na^+^, and K^+^) in positive ion mode by using DHB as a matrix. MALDI imaging of saturated hydrocarbons (HCs) however, is more challenging because these species do not contain any polar groups neither susceptible to protonation nor to which cations or anions can be easily attached (Cvacka and Svatos, 2003). When monovalent cations of transition metals (e.g., Fe, Mn, Cu) are co-deposited on MALDI target with HCs, they give cationized species which can be detected in a mass spectrometer. However, due to the high reactivity of transition metals, HCs are easily fragmented during analysis, hampering molecular identification (Cvacka and Svatos, 2003). The reactivity of silver with HCs is lower than that of any other transition metals so it can be used to generate intact silver adduct ions. MALDI imaging of epicuticular wax in *Arabidopsis* has been successfully reported by using silver colloid as matrix. In this study, 14 cuticular wax compounds were identified in *Arabidopsis* wild-type (Ler) and genetic stock CS8 (which carries carried the mutant alleles *cer2-2, ap2-1*, and *bp1*) leaves (Cha et al., 2009). The pitfall of silver matrix is that silver is present with similar abundance for its two stable isotopes. Each molecule, then, produces a group of silver adduct ion peaks with two major ions [monoisotopic mass of the metabolite + ^107^Ag or ^109^Ag]^+^, making compound identification and quantification difficult (Cvacka and Svatos, 2003; Cha et al., 2009). Reports on MALDI imaging of saturated wax esters in *Arabidopsis* and date palm leaves suggested that the lithium salt of DHB (LiDHB) is the most versatile matrix for detection of a majority of neutral lipids and it can potentially replace the currently used silver salts (Vrkoslav et al., 2010).

Localization of lipids is the most frequent application of DESI imaging in mammalian tissues, while DESI imaging of lipids in plants has not yet been reported. Since lipids are more readily ionized by DESI, DESI would be an ideal complementary tool to MALDI for mapping lipids in plants where high spatial resolution is not required. Mixtures of water-methanol or water-acetonitrile, with or without an acidic modifier are the most commonly used spray solvents for DESI imaging of lipids (Eberlin et al., 2011).

### Proteins and peptides

The fundamental component of a protein is the polypeptide chain composed of amino acid residues. Proteins are highly ubiquitous either as plant storage proteins (e.g., legumin, vicilin, convicilin, albumin, and gliadin) or as functional proteins such as enzymes, membrane components or hormones (Aluko, 2015).

MSI studies of proteins and peptides are in general particularly challenging, and, up to now, they are well described in mammalian tissues. Sample preparation is the first critical point for this type of studies and it is more challenging than for other molecules. The different protocols include several additional washing steps to remove endogenous molecular species, such as salts and sugars, which may interfere with protein desorption/ionization efficiency, to ensure tissue dehydration and fixation, and to prevent proteolysis (Schwartz et al., 2003). The wash procedure varies in solvent composition, temperature and duration depending on the tissue, and it needs to be optimized accordingly. Even after careful optimization, proteins larger than 25kDa are not routinely detectable by MALDI MSI, as they are not efficiently stabilized in the matrix solution and are not extractable from the tissue (Franck et al., 2010). On-tissue digestion of large proteins can be used to detect and identify larger proteins, but the treatment with proteolytic enzymes enhances analyte diffusion thus reducing the spatial resolution in MSI studies (Kaspar et al., 2011). In the specific case of plants, protein identification is also challenging, due to the lack of extensive and reliable databases (Kaspar et al., 2011).

The application of MSI of proteins in plants has, however, been illustrated in few cases, as demonstrated by the MALDI imaging analysis of proteins in developing barley grains (Kaspar et al., 2011) and in soybean cotyledons (Grassl et al., 2011), where tissue-specific protein expression patterns have been revealed. A detailed analytical protocol is presented and discussed in the latter example, there sinapinic acid (SA) is suggested as matrix for MALDI imaging of proteins (>3000 Da), and CHCA and DHB for peptides (< 3000 Da).

### Terpenoids

Terpenoids are a large and diverse class of metabolites which are built up from isoprene. Plants employ terpenoids for a variety of functions in growth and development but use the majority of them for more specialized chemical interactions and protection in the abiotic and biotic environment (Tholl, 2015).

Distribution of terpenoids in *Hypericum perforatum* have been studied by LDI imaging on fresh tissues (Holscher et al., 2009) and by DESI imaging on leaves and their imprints (Thunig et al., 2011) respectively. The results of the two studies are in complete agreement showing that hyperforin and adhyperforin are found in translucent glands, and hypericin, pseudohypericin, protopseudohypericin, and protohypericin are exclusively located in dark glands in leaves. Since they are highly UV absorbing compounds, application of a matrix is not necessary and thereby LDI allows 10 μm spatial resolution with a 10 × 10 μm laser focus setting, removing the spatial limitations associated with the use of a matrix. It is important to point out that the understanding of the biological function of natural products requires direct fine-scale evaluation in the tissue of the producing organism (Esquenazi et al., 2009). One example is the DESI imaging of a tropical red alga tissue surface where bromophycolide A and B are found exclusively distributed in association with distinct surface patches at concentrations sufficient to inhibit the detrimental *Lindra thalassiae* fungus (Lane et al., 2009).

### Alkaloids

Alkaloids contain secondary, tertiary, or quaternary nitrogen atoms. In plants, they act as defense compounds against pathogenic organisms and herbivores, but they can also be used as protoxins by insects which further modify them before incorporating them into their own defense system or secretions (Ghosh, 2000). Alkaloids are typically found in particular medicinal plants organs, often defined as a “medicinal part” (Shitan and Yazaki, 2007). Knowing their localization is valuable not only to understand their metabolic origins, but also to optimize their isolation process from the compound containing parts.

Rapid profiling of alkaloids has been performed in several plant species by DESI and MALDI. Relevant examples include DESI profiling of alkaloids in *Conium maculatum, Datura stramonium*, and *Atropa belladonna* (Talaty et al., 2005), and MALDI profiling of alkaloids in *Rhizoma Coptidis* and *Strychnos nux-vomica L.* (Lu et al., 2010). This type of studies provided the basic protocols for MS imaging of alkaloids in plants. Distribution of alkaloids has been mapped for several purposes: for example MALDI imaging of capsaicin in capsicum fruits (Taira et al., 2012) and DESI imaging of malabaricone C. in *Myristica malabarica* seed (Ifa et al., 2011) to study their metabolic origin, and MALDI imaging of fruiting bodies of a mushroom to screen for new compounds (Jaeger et al., 2013). All the above examples were performed in positive ion mode, and alkaloids are mostly detected as [M+H]^+^, and in some cases as salt adducts, such as [M+K]^+^.

### Phenolics

Phenolics are compounds possessing one or more aromatic rings with one or more hydroxyl groups. Plant phenolics include phenolics acids, flavonoids, tannins and the less common stilbenes and lignans. They are generally involved in defense against ultraviolet radiation or aggression by pathogens, parasites and predators, as well as contributing to plants' colors (Dai and Mumper, 2010).

Distribution of phenolics has been mapped in several plants including strawberry (Zhang et al., 2007), grapevine leaf (Becker et al., 2014), apple (Zhang et al., 2007; Franceschi et al., 2012), *Arabidopsis* (Cha et al., 2008; Holscher et al., 2009), rice (Yoshimura et al., 2012b), licorice (Li et al., 2014a) and blueberry (Yoshimura et al., 2012a) by MALDI, GALDI, and LDI imaging. Flavonols are mainly detected as [M-H]^−^ in negative ion mode, while anthocyanins are mostly detected in positive mode as [M]^+^. Both CHCA and DHB are common matrices used for the analysis of phenolics in MALDI imaging. DESI imaging was recently applied to localize flavonols in ginkgo leaves in negative ionization mode, and anthocyanins in strawberry in positive mode (Cabral et al., 2013). Information on their distribution has been used to explain gene expression patterns in the wild-type and mutant (*transparent testa*; *tt7*) *Arabidopsis* flowers. In particular, the *Arabidopsis tt7* mutant effectively blocks the production of quercetin, isorhamnetin and their glycoside derivatives, but leads to the accumulation of kaempferol and its glycoside glycosides. By imaging the distribution of several flavonoids in wild-type and the *tt7* mutant flowers, the MSI results could explain the expression of the *TT7* gene localization to the proximal part of the petal and the expression of the other genes of the upstream pathway that are evenly expressed throughout the petal (Korte et al., 2012).

### Elements

The mapping of the biologically essential trace metals (i.e., Cu, Zn, Fe), metalloids (i.e., Se), or non-metals (i.e., S, P, I) is of increasing interest in modern bio-analytics (Becker et al., 2010). LA-ICP-MS and SIMS are two major MSI ion sources of choice for trace elements localization (Heard et al., 2002; Zhu et al., 2012; Choi et al., 2014; Hanć et al., 2014). In particular, the absence of charging-up effects combined with fewer matrix effects makes LA-ICP-MS a simple quantification tool in which is possible to apply certified standard reference materials or matrix-matched laboratory standards (Becker et al., 2010). For example, with the apple leaf SRM NIST 1515 as a certified standard reference material, distribution of K, P, Mg, and Mn in *Elsholtzia splendens* leaves has been quantitatively determined employing LA-ICP-MS imaging. The results showed that the four elements were predominantly located in the leaf veins, which highlighted the importance of the vein in transporting macro-essential elements (Becker et al., 2008). Other attractive feature of SIMS and LA-ICP-MS is that they require little or no sample preparation.

## Conclusion

The goal and philosophy of sample handling in MSI is to preserve the integrity of the tissue samples, keeping the original localization of the analytes within the tissues, and increasing the amount of ions desorbed from the sample surface. The importance of sample preparation for plants has been highlighted in several recent excellent reviews (Fujimura and Miura, 2014; Horn and Chapman, 2014; Spengler, 2014; Boughton et al., 2015; Sturtevant et al., 2016). A number of factors are reported to be critical for successful sample preparation in MSI, and they range from tissue storage, sectioning and mounting to the selection of the optimal ionization aiding treatment. It is worth noting that each factor should be carefully optimized depending on the characteristics of the MSI instrument, the nature of the sample and the analytes of interest.

Among the different aspects, it is important to remark that further improvement in reproducibility in sample preparation is highly needed in order to allow reliable inter-sample comparison, and to improve serial-section based 3D MSI. In this regard, the setting-up of simplified and automatic sample preparation pipelines is required.

In addition, we believe that further development of sample preparation strategies able to account for tissue-specific ion suppression (i.e., local chemical and morphological suppression) is needed in order to increase molecular coverage and improve the quantitative potential of MSI.

It is anticipated that these advances in sample preparation will largely expand the potential of MSI in plant sciences.

## Author contributions

Conceptualization, DY and FP; Methodology, DY, FP, and AA; Investigation, DY, MS, and RI; Writing—Original Draft, DY; Writing-Review and Editing, FP, LB, AA, MS, RI, KF, and SA; Visualization, DY and LB; Funding Acquisition, FP and AA; Supervision, FP and AA.

### Conflict of interest statement

The authors declare that the research was conducted in the absence of any commercial or financial relationships that could be construed as a potential conflict of interest.
